# MicroRNA-196a regulates bovine newborn ovary homeobox gene (NOBOX) expression during early embryogenesis

**DOI:** 10.1186/1471-213X-11-25

**Published:** 2011-05-06

**Authors:** Swamy K Tripurani, Kyung-Bon Lee, Gabbine Wee, George W Smith, Jianbo Yao

**Affiliations:** 1 Laboratory of Animal Biotechnology and Genomics, Division of Animal and Nutritional Sciences, West Virginia University, Morgantown, WV 26506, USA; 2 Laboratory of Mammalian Reproductive Biology and Genomics, Department of Animal Science, Michigan State University, East Lansing, MI 48824, USA; 3 Department of Physiology, Michigan State University, East Lansing, MI 48824, USA

**Keywords:** NOBOX, bovine, oocyte, early embryogenesis, microRNA, maternal to zygotic transition

## Abstract

**Background:**

Oocyte-derived maternal RNAs drive early embryogenesis when the newly formed embryo is transcriptionally inactive. Recent studies in zebrafish have identified the role of microRNAs during the maternal-to-embryonic transition (MET). MicroRNAs are short RNAs that bind to the 3' UTR of target mRNAs to repress their translation and accelerate their decay. Newborn ovary homeobox gene (NOBOX) is a transcription factor that is preferentially expressed in oocytes and essential for folliculogenesis in mice. NOBOX knockout mice are infertile and lack of NOBOX disrupts expression of many germ-cell specific genes and microRNAs. We recently reported the cloning and expression of bovine NOBOX during early embryonic development and our gene knockdown studies indicate that NOBOX is a maternal effect gene essential for early embryonic development. As NOBOX is a maternal transcript critical for development and NOBOX is depleted during early embryogenesis, we hypothesized that NOBOX is targeted by microRNAs for silencing and/or degradation.

**Results:**

Using an algorithm "MicroInspector", a potential microRNA recognition element (MRE) for miR-196a was identified in the 3' UTR of the bovine NOBOX mRNA. Expression analysis of miR-196a in bovine oocytes and during early embryonic development indicated that it is expressed both in oocytes and embryos and tends to increase at the four-cell and eight-cell stages. Ectopic expression of NOBOX and miR-196a in HeLa cells inhibited the expression of NOBOX protein compared to the control cells without miR-196a. Similarly, the activity of a luciferase construct containing the entire 3' UTR of bovine NOBOX was suppressed, and the regulation was abolished by mutations in the miR-196a binding site indicating that the predicted MRE is critical for the direct and specific binding of miR-196a to the NOBOX mRNA. Furthermore, ectopic expression of miR-196a mimic in bovine early embryos significantly reduced the NOBOX expression at the both mRNA and protein levels.

**Conclusion:**

Collectively, our results demonstrate that miR-196a is a bona fide negative regulator of NOBOX during bovine early embryogenesis.

## Background

The earliest stages of embryonic development in vertebrates primarily rely on the maternal RNA and proteins synthesized during oogenesis [1,2]. The period of maternal control of embryonic development varies among species according to the onset of embryonic genome activation and the degradation of maternal gene products [3]. The major onset of embryonic genome activation begins during the two-cell stage in mice; the four-cell stage in humans, rats and pigs, and during the eight-cell to 16-cell stage in cattle and sheep [4]. Upon fertilization, in mouse embryos, 90 percent of the maternal mRNA is degraded by the two-cell stage, coincident with the complete activation of the embryonic genome [5,6]. There is direct evidence that maternal mRNA clearance is critical for early embryonic development. For example oocyte-specific c-mos mRNA, essential for regulating meiotic arrest at metaphase, is degraded soon after fertilization and injection of c-mos protein into Xenopus two-cell embryos induces cleavage arrest [7]. In mouse, maternal mRNA degradation is dependent on the 3' untranslated region (3' UTR) of the mRNA transcript. For example, chimeric mRNAs composed of the c-mos coding region fused to the hypoxanthine phosphoribosyltransferase (Hprt) 3' UTR have reduced rates of degradation following microinjection into mouse fertilized oocytes [8]. Thus degradation of maternal mRNAs is critical to embryogenesis and represents a conserved mechanism of vertebrate development.

Multiple negative regulatory mechanisms are critical for post-transcriptional regulation of maternal transcripts, such as transcript deadenylation and interaction with RNA-binding proteins in a nonspecific or sequence-specific fashion [9]. Recent studies in zebrafish have established a role for microRNAs (miRNA) as key regulatory molecules targeting maternal mRNA for degradation during the maternal-to-embryonic transition (MET) [10]. MicroRNAs are endogenous small noncoding RNAs that bind primarily to the 3' UTR of target mRNAs to repress their translation and accelerate their decay [11]. The majority of miRNAs are evolutionarily conserved across species boundaries and play essential roles in regulating many distinct processes such as animal development and growth, cell differentiation, signal transduction, cancer, disease, virus immune defense, programmed cell death, insulin secretion and metabolism [12-14].

In recent years, several studies have revealed the significance of miRNAs in reproduction and embryonic development. For example, targeted disruption of Dicer, a key enzyme involved in miRNA processing and the synthesis of small interfering RNAs from long double-stranded RNA [15,16] in mice and zebrafish resulted in embryonic lethality due to abnormalities in morphogenesis, cell division and chromosome organization [17-21]. In zebrafish, miR-430 has been linked to maternal mRNA decay accompanying the maternal-to-embryonic transition [10]. At the onset of embryonic genome activation, the level of miR-430 substantially increases and the miRNA targets several hundred maternally provided mRNAs by binding to the complementary sites in their 3' UTR and promotes their deadenylation [10]. Furthermore, miR-196a regulates mammalian development via targeting homoeobox clusters [22] and misexpression of miR-196a leads to specific eye anomalies in a dose-dependent manner in Xenopus laevis [23].

Newborn ovary homeobox gene (NOBOX) is a transcription factor, identified by *in silico *subtraction of expressed sequence tags (ESTs) derived from newborn ovaries in mice [24]. NOBOX mRNA and protein are preferentially expressed in oocytes throughout folliculogenesis [25]. Nobox knockout mice are infertile due to disrupted folliculogenesis and expression of many germ-cell specific genes and miRNAs is perturbed in such animals [25,26]. Furthermore, mutations in the NOBOX gene associated with premature ovarian failure have been described in humans [27,28]. We recently established a key role for NOBOX in bovine early embryonic development [29]. Bovine NOBOX is stage-specifically expressed during oocyte maturation and early embryonic development and of maternal origin. Depletion of NOBOX in bovine zygotes by siRNA microinjection impaired embryo development to the blastocyst stage. Furthermore, knockdown of NOBOX affected the expression of genes from the embryonic genome critical to early development and expression of pluripotency genes was altered in the inner cell mass of NOBOX siRNA injected embryos that reached the blastocyst stage. However, despite its established role in folliculogenesis and early embryonic development, the post-transcriptional regulation of NOBOX has not been investigated. Given the importance of NOBOX, as a maternal transcript critical for development, and observed depletion of NOBOX during MET, we hypothesized that NOBOX is targeted by miRNAs for silencing and/or degradation in early embryos. In this study we identified a miRNA (miR-196a) targeting bovine NOBOX, examined the temporal expression of miR-196a during bovine early embryonic development and determined the effect and specificity of miR-196a in regulating bovine NOBOX expression both exogenously (HeLa cells) and endogenously in early embryos.

## Results and Discussion

### miR-196a binds to the 3' UTR of bovine NOBOX

**Figure 1:**
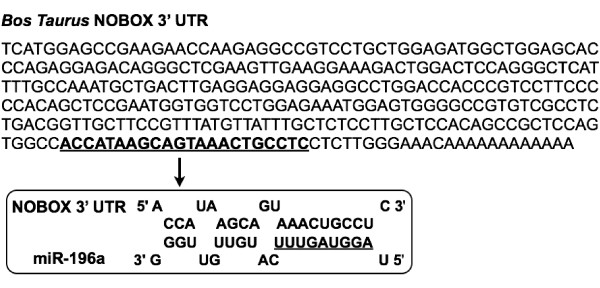
**Prediction of a miR-196a binding site in the 3' UTR of bovine NOBOX mRNA**. The predicted miR-196a binding site is underlined.

MicroRNAs regulate mRNA translation rate by perfect or imperfect base pairing with the 3' UTR regions of their targets [30]. It has been predicted that one miRNA can potentially regulate translation of up to a hundred mRNAs, which creates a challenge for experimentally validating miRNA-specific targets [31]. To identify miRNAs that potentially regulate NOBOX expression, we analyzed the 3' UTR sequence of bovine NOBOX using the "Microinspector" algorithm to predict potential miRNA target sites [32]. miR-196a was chosen for further studies, because the predicted MRE in the bovine NOBOX 3' UTR had a low predicted free energy of hybridization with the cognate miRNA (-19.8 kcal/mol), suggesting a stable miRNA: mRNA duplex within the 9 nucleotide (nt) seed region at the 5' end of the miRNA (Figure 1). This seed sequence is an important determinant of miRNA-induced repression of gene expression [33]. RNA secondary structure prediction analysis using Mfold [34] revealed that the apparent miR-196a binding site was positioned on a hairpin-loop structure, in an exposed position, which might facilitate miRNA accessibility. In addition, when the NOBOX sequence was analyzed with other miRNA target prediction algorithms, miR-196a always was listed as a top candidate miRNA, further indicating that miRNA-196a might be a potential post-transcriptional regulator of NOBOX in early embryos. The lack of conservation of miR-196a recognition sequence in bovine NOBOX might be due to the rapid drifting of 3' UTR during evolution [31,35]. Furthermore, it has been reported that a large fraction of bona fide targets of microRNA would be missed [10,31,36] if evolutionary conservation were used as the sole criterion for predicting targets. Moreover, recent studies support a functional role for this specific miRNA as miR-196a targets specific homeobox genes (HoxB8, HoxC8, HoxD8 and HoxA7) in mouse embryos and mammalian cells and plays a major role in animal development [22]. Thus, the functional role of miR-196a in regulation of NOBOX was further investigated.

### miR-196a is spatio-temporally regulated during development

**Figure 2:**
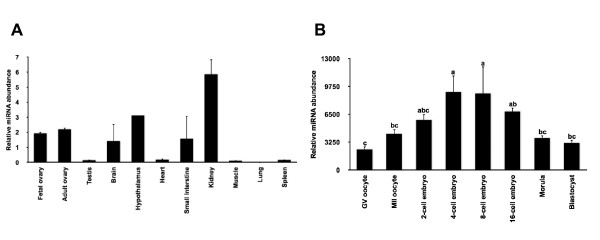
**Spatial and temporal expression profile of miR-196a**. (A) Tissue distribution of miR-196a analyzed by quantitative real-time PCR. Quantity of miRNA-196a was normalized to abundance of RPS18 mRNA and abundance expressed as relative fold change using the sample with the lowest value as the calibrator (n = 4 per tissue; mean ± SEM depicted). (B) Relative abundance of miR-196a in bovine oocytes and *in vitro *produced bovine early embryos (n = 4 pools of five oocytes/embryos each). Quantity of miRNA was normalized relative to abundance of miR-125b. The relative amount of miR-196a was expressed as relative fold change using the sample with the lowest value as the calibrator (n = 4, mean ± SEM). Different letters indicate statistical difference (P < 0.05).

To determine the tissue specific expression pattern of miR-196a, quantitative real-time PCR was performed. As shown in Figure 2A, miR-196a is expressed predominantly in kidney; it is also detected significantly in fetal and adult ovary, brain and hypothalamus. A similar expression pattern was observed in mice where miR-196a is enriched in the kidney and adult reproductive tissues [37]. In order to examine if miR-196a expression is inversely correlated to bovine NOBOX expression during early embryonic development, we analyzed miR-196 expression during oocyte maturation and early embryogenesis. Expression analysis indicates that bovine miR-196a is increased in four-cell and eight-cell stage embryos relative to germinal vesicle stage oocytes and declines at morula and blastocyst stages (Figure 2B). The increased expression level of miR-196a near the eight-cell stage of embryogenesis potentially indicates miR-196a involvement in maternal transcript degradation during the maternal-to-zygotic transition, as was observed for miR-430 in zebrafish [10] miR-427 in Xenopus [38] and miR-290 in mouse [20]. Moreover, when the spatio-temporal expression pattern of miR-196a is compared with the expression pattern of bovine NOBOX during early embryogenesis, miR-196a expression increases steadily from two-cell to eight-cell stage of embryogenesis, while NOBOX expression decreases gradually during the same period [29]. Thus, the inverse relationship between miR-196a and NOBOX expression/activity supports the proposed role of miR-196a as a physiological regulator of NOBOX during early embryogenesis.

### miR-196a specifically suppresses the expression of bovine NOBOX

**Figure 3:**
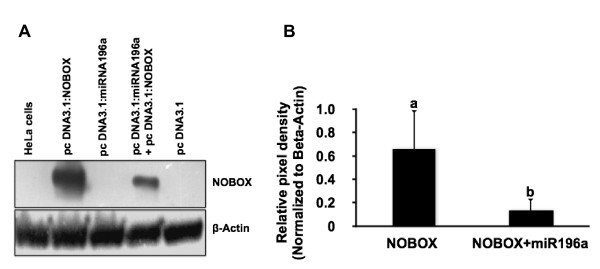
**Regulation of bovine NOBOX expression by miR-196a *in vitro *in HeLa cells**. (A) Representative Western blot showing specific suppression of bovine NOBOX by miR-196a in HeLa cells. β-Actin was used as loading control. The experiment was repeated four times with similar results, and a representative experiment is shown. (B) Semi-quantitative analysis of miRNA-196a regulation of NOBOX expression in transfected HeLa cells. Abundance of NOBOX protein in each sample was determined by densitometry and normalized relative to abundance of β-Actin protein (control). Data are expressed as mean relative pixel density (n = 4 mean ± SEM). Different letters indicate statistical difference (P < 0.05).

To confirm the binding of miR-196a to bovine NOBOX *in vitro*, HeLa cell transfection studies were conducted. A significant inhibition of NOBOX expression was observed in HeLa cells ectopically expressing both NOBOX and miR-196a (Figure 3A) relative to cells transfected with NOBOX alone. Semi-quantitative analysis of western blot data showed a significant inhibition of NOBOX expression in the miR-196a-transfected cells (Figure 3B). These results unequivocally show that bovine NOBOX is regulated at the post-transcriptional level by miR-196a and further supports the hypothesis that miR-196a is responsible for the negative regulation of NOBOX.

**Figure 4:**
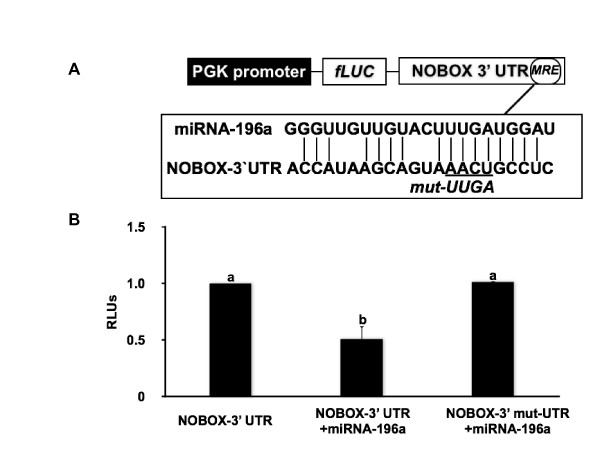
**miR-196a specifically binds to the 3' UTR of bovine NOBOX and regulates its expression**. (A) Schematic of the luciferase reporter constructs used to demonstrate sequence specificity in recognition sequence in bovine NOBOX 3' UTR mediating miR-196a mediated repression. Nucleotides changed to generate the target site mutant 3' UTR are underlined (B) Repression of luciferase activity due to specific interaction between miR-196a and the predicted MRE in the luciferase-NOBOX-3' UTR constructs. Repression of luciferase reporter gene activity by miR-196a was abolished when the MRE was mutated. Data is presented as relative firefly luciferase units (RLUs). Relative firefly luciferase values were determined by a ratio of firefly to renilla luciferase with the negative control (cells transfected with native NOBOX-3' UTR construct alone) set at 1. Each group represents the mean ± SEM of four wells for an experiment repeated four times with similar results. Different letters indicate statistical difference (P < 0.05).

Furthermore, luciferase reporter assays were performed to validate specificity of the miR-196a regulation of NOBOX through the predicted miR-196a recognition sequence in the 3' UTR of NOBOX. NOBOX 3' UTR sequence was inserted downstream of the firefly luciferase coding region. Mutations in the predicted MRE in the 3' UTR of the NOBOX for miR-196a were created such that interaction between miR-196a and NOBOX is compromised (Figure 4A). Ectopic expression of miR-196a by transfection of miR-196a duplex into the HeLa cells suppressed activity of a chimeric luciferase construct containing the miR-196a MRE of NOBOX at its 3' end (Figure 4B). Luciferase activity was restored when a four-base mismatch mutation was introduced into the seed region of the miRNA-196a recognition sequence in the NOBOX 3' UTR (Figure 4B). These data indicate the predicted MRE is critical for the direct and specific binding of miR-196a to NOBOX transcript.

### miR-196a represses endogenous NOBOX in bovine early embryos

**Figure 5:**
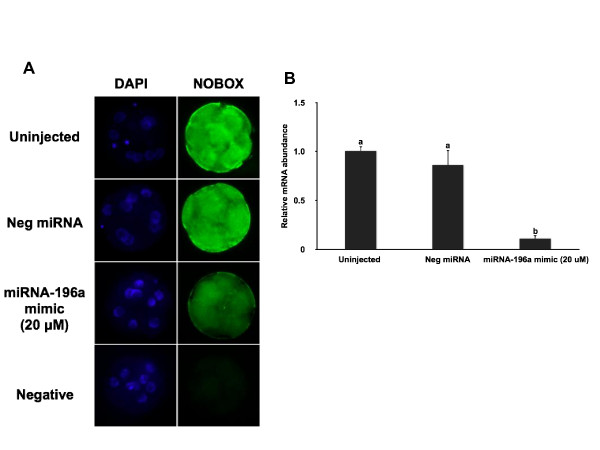
**Microinjection of miR-196a mimic represses endogenous NOBOX expression in bovine early embryos**. (A) Effect of miR-196a mimic microinjection on abundance of NOBOX protein in 8-cell stage embryos as determined by immunocytochemical analysis using confocal spinning-disk microscopy (n = 4 pools of 5-10 embryos per treatment). Uninjected embryos and embryos injected with a nonspecific miRNA (Neg miRNA) were used as controls. Nuclear DNA was stained with DAPI. (B) Effect of miR-196a mimic microinjection on abundance of NOBOX mRNA in eight-cell embryos as determined by real-time PCR. Data were normalized relative to abundance of endogenous control ribosomal protein S18 (RSP18) and are shown as mean ± SEM (n = 4 pools of 10 embryos per treatment). Different letters indicate statistical difference (P < 0.05).

Since we determined in heterologous systems that miR-196a is capable of regulating NOBOX expression through direct binding to the 3' UTR of its mRNA, the ability of miR-196a to regulate endogenous NOBOX expression in early embryos was determined. Microinjection of miRNA mimics into zygotes has been utilized previously as a tool to determine effects of overexpression of specific miRNAs in mouse and zebrafish embryos [39-41]. Ectopic expression of miR-196a mimic in bovine embryos effectively reduced NOBOX protein expression in eight-cell embryos compared to uninjected and the negative control miRNA-injected embryos (Figure 5A). Furthermore, recent studies have suggested that miRNAs not only inhibit productive translation but also accelerates target mRNA decay [42,43]. Microinjection of miR-196a mimic in bovine embryos significantly reduced NOBOX mRNA levels in eight-cell embryos by more than 80% relative to uninjected and negative control miRNA-injected embryos (Figure 5B).

The degradation of the untranslated maternal RNA pool is very critical to early embryonic development [1]. The translation potential of a maternal mRNA transcript is affected by the length of the poly (A) tail as it confers mRNA stability and stimulates translation via interaction of poly (A) binding protein (PABP) with the 5' m7G cap [44,45]. Moreover, maternal mRNAs are dependent on post-transcriptional and post-translational mechanisms to regulate their activity, as they cannot be repressed at the transcriptional level [9,46]. Recent studies in zebrafish and Xenopus found that miRNAs promote deadenylation of target mRNAs and induce maternal mRNA degradation/clearance during early embryogenesis [10,38], indicating that miRNA-induced clearance of maternal mRNAs might be a universal mechanism during MET. Thus, a similar mechanism is likely to be involved in the miR-196a negative regulation of NOBOX expression in bovine embryos during MET.

miR-196a is an evolutionary conserved miRNA that has been identified in a wide range of vertebrate species. It is expressed from intergenic regions of HOX gene clusters, and targets several HOX genes in these clusters, which are known to play crucial roles during development [22,47,48]. Recent studies showed that 75% of tumors express high levels of miR-196a and miR-196a is involved in regulating key pathways such as AKT signaling, p53 and WNT signaling pathways [49,50]. It has also been reported that miR-196a is differently regulated during polycystic kidney disease suggesting that miR-196 is important for normal functioning of kidney [51]. The involvement of miR-196a in regulating the expression of NOBOX supports a new role of this miRNA in early embryonic development during MET.

## Conclusions

Collectively, our results demonstrate the ability of miR-196a to negatively regulate NOBOX expression in a sequence specific fashion and the ability of miR-196a to suppress NOBOX mRNA and protein in early embryos. Future studies of interest will investigate whether loss of miR-196a has any effect on the early embryonic development and identify putative miR-196a targets by next generation sequencing analysis of miR-196a depleted and wild type embryos.

## Methods

### Bioinformatics Analysis

To examine the possibility of NOBOX regulation by miRNAs, we searched for potential microRNA recognition elements (MRE) in the NOBOX 3' UTR using Microinspector http://bioinfo.uni-plovdiv.bg/microinspector/, an algorithm for detection of possible interactions between miRNAs and target mRNA sequences [32].

### Tissue collection, RNA isolation and microRNA expression analysis

Bovine tissue sample collection, total RNA isolation and miRNA expression analysis in multiple tissues, oocytes and early embryos were performed as described previously [52].

### Plasmid construction

The full-length bovine NOBOX mRNA sequence was amplified from bovine adult ovary cDNA samples by PCR using gene-specific primers containing restriction sites BamHI/XhoI (Additional file 1, Table 1 for the list of primer sequences). The PCR product was digested with BamHI and XhoI enzymes and subsequently cloned into pcDNA3.1 (Invitrogen, Carlsbad, CA) vector digested with the same enzymes. pcDNA3.1: miRNA196a was constructed by PCR amplifying a ~220nt region of genomic sequence surrounding pre-miR-196a from bovine genomic DNA sample using primers containing restriction sites BamHI/XhoI (Additional file 1, Table 1 for the list of primer sequences). The PCR product was digested and subsequently cloned into pcDNA3.1 vector digested with BamHI and XhoI. For construction of a vector containing NOBOX-3' UTR fused to the 3' end of a luciferase reporter, we used the dual luciferase pmirGLO vector (Promega, Madison, WI). The NOBOX 3' UTR was amplified from pcDNA3.1: NOBOX construct using primers containing restriction sites SacI/XbaI (Additional file 1, Table 1 for the list of primer sequences). The PCR product was digested with SacI and XbaI and subsequently cloned into dual luciferase pmirGLO vector digested with the same enzymes. Mutation of the mir-196a miRNA recognition element (MRE) in the NOBOX 3' UTR was performed using the QuickChange site-directed mutagenesis kit (Stratagene, Santaclara, CA) according to the manufacturer's instructions. (Additional file 1, Table 1 for the list of primer sequences).

### Cell culture and Reporter assay

HeLa cells were cultured in DMEM (Invitrogen, Carlsbad, CA) containing 10% FBS and 1% penicillin/streptomycin (Invitrogen, Carlsbad, CA). For transient transfection, FuGENE6 (Roche Applied Science, Indianapolis, IN) was used according to manufacturer's instructions. Following transfection, cells were incubated for 48 h before harvest for western blotting and luciferase assay. Luciferase assay was performed using the Dual-Glo luciferase assay system (Promega, Madison, WI) as described by the manufacturer. Firefly luciferase activity was normalized to renilla luciferase activity to adjust for variations in transfection efficiency among experiments. All transfection experiments were performed in quadruplicate (n = 4) with data averaged from four independent experiments.

### Western blot analysis

Western blot was performed as previously described [53] with minor modifications. After 48 h of transfection, HeLa cell lysates were harvested and washed once with phosphate-buffered saline (PBS), suspended in 50 μl of PBS, and mixed with an equal volume of Laemmli sample buffer (Bio-Rad, Hercules, CA). Protein samples (15 μg/each) were separated on a 4-20% gradient polyacrylamide gel (Bio-Rad, Hercules, CA) and electroblotted onto a polyvinylindene difluoride (PVDF) membrane (Bio-Rad, Hercules, CA). Following transfer and blocking in 5% nonfat dry milk in Tris-buffered saline containing 0.1% Tween-20 (TBST) for one hour, the membrane was then incubated in NOBOX antibody (ab41612; Abcam, Cambridge, MA) diluted 1:100 in blocking buffer overnight at 4°C. After washing three times with TBST, the membrane was incubated for 1 h with horseradish peroxidase-conjugated goat anti-rabbit IgG (Pierce, Rockford, IL) diluted 1:10 000 in blocking solution. The membrane was washed again with TBST, followed by detection with SuperSignal West Pico Chemiluminescent Substrate (Pierce, Rockford, IL). The membrane was stripped in Restore Plus Western Blot Stripping Buffer (Pierce, Rockford, IL), followed by detection of β-actin (ACTB) protein (positive control) using anti-β-actin antibody (Ambion, Austin, TX) and horseradish peroxidase-conjugated goat anti-mouse IgG (Pierce, Rockford, IL).

### Microinjection experiments

Procedures for in vitro maturation of oocytes (obtained from abattoir-derived ovaries) and in vitro fertilization to generate zygotes for microinjection and for subsequent embryo culture were conducted basically as described [54,55]. Presumptive zygotes collected at 16-18 hours post-fertilization (hpf) were used in all microinjection experiments. Mature miRNA-196a mimic (MIMAT0000226) and negative control cel-miR-67 (CN-001000-01-05) were obtained from Dharmacon Technologies (Dharmacon Inc, Lafayette, CO), and diluted with RNase free water to a final concentration of 10 μM and 20 μM before microinjection (The final concentration used for microinjection was 20 μM based on initial experiments showing this concentration is more effective in repressing Nobox expression). Approximately 20 pl of miRNA mimic (20 μM) was injected into the cytoplasm of zygotes using an inverted Nikon microscope equipped with micromanipulators (Narishige International USA, Inc., East Meadow, NY). Uninjected embryos and embryos injected with above negative control miRNA were used as control groups. Each group contained 25-30 embryos per replicate (n = 4). After microinjection, groups of embryos were cultured in 75- to 90-μl drops of potassium simplex optimization medium (KSOM) (Specialty Media, Phillipsburg, NJ) supplemented with 0.3% bovine serum albumin (BSA) until 72 h after insemination at which time point embryos were collected. The efficiency of NOBOX mRNA/protein knockdown in miRNA-196a mimic injected and control embryos was determined by quantitative real-time PCR analysis and immunocytochemistry in eight-cell stage embryos as described previously [30]. Imaging was performed using confocal spinning-disk microscopy. Optical sections every 1 μm were acquired for each embryo and MetaMorph software (Universal Imaging, Downingtown, PA, USA) was used for image acquisition and analysis.

### Statistical Analysis

One-way ANOVA using the general linear models (GLM) procedure of SAS were used to determine the significance of differences in mRNA abundance and between the treated samples and the controls where values resulted from the luciferase reporter assay, quantitative real-time PCR and western blots. Different letters indicate significant differences (P < 0.05).

## Authors' contributions

SKT designed and performed most of the experiments including expression analysis of miRNA, preparation of constructs, cell transfection and analysis of gene expression. SKT also drafted the manuscript. KBL and GW performed the microinjection experiments. GWS and JY designed the study and supervised the experimental work. All authors read and approved the final manuscript.

## Supplementary Material

Additional file 1 **Table 1. List of primers used in this study**. Click here for file
